# Bioavailability of Macro- and Microelements in Rats Fed Hypercholesterolemic Diets Containing *Actinidia arguta* Fruits

**DOI:** 10.3390/foods11111633

**Published:** 2022-06-01

**Authors:** Mikołaj Antoni Gralak, Iwona Lasocka, Maria Leontowicz, Hanna Leontowicz, Piotr Latocha, Shela Gorinstein

**Affiliations:** 1 Departament of Physiological Sciences, Warsaw University of Life Sciences—SGGW, 02-787 Warsaw, Poland; maria_leontowicz@sggw.edu.pl (M.L.); hanna_leontowicz@sggw.edu.pl (H.L.); 2 Departament of Biology of Animal Environment, Warsaw University of Life Sciences—SGGW, 02-787 Warsaw, Poland; iwona_lasocka@sggw.edu.pl; 3 Department of Environmental Protection and Dendrology, Warsaw University of Life Sciences—SGGW, 02-787 Warsaw, Poland; piotr_latocha@sggw.edu.pl; 4 Institute for Drug Research, School of Pharmacy, Faculty of Medicine, The Hebrew University of Jerusalem, Jerusalem 9112001, Israel; shela.gorin@mail.huji.ac.il

**Keywords:** kiwiberry, *Actinidia arguta*, macroelements, trace elements, bioavailability

## Abstract

The aim of this study was to estimate the influence of different cultivars of *Actinidia arguta* (kiwiberry) on the bioavailability of mineral elements and to examine the mineral profile of rats fed atherogenic diets enriched with kiwiberries. The following cultivars of *Actinidia arguta* were used: Bingo, M1, Anna, Weiki, Jumbo, and Geneva. Kiwiberry has recently become popular in the market. It is a precious source of biologically active components, vitamins, and minerals. The livers, spleens, and kidneys were examined for mineral contents using the flame atomic absorption spectroscopy method. The bioavailability of Ca, Mg, Fe, Mn, Zn, and Cu was evaluated. The addition of kiwiberries in atherogenic diets increased the contents of Fe in the rat liver. The bioavailability of Mn, Zn, and Cu, calculated on the basis of the contents in the livers, was significantly decreased in rats fed diets with 5% additional kiwiberries. We supposed that the effect of kiwiberry on the bioavailability of the studied minerals may be related to the diet components of bioactive substances present in fruits (polyphenols, vitamins, dietary fiber, and tannins).

## 1. Introduction

The *Actinidia arguta* (Siebold et Zucc.) Planch. ex Miq. fruit, also called hardy kiwifruit, kiwiberry, or mini kiwifruit, is a new product on the market [1,2]. It belongs to *Actinidia* genus and is not as popular as its bigger cousin, the kiwifruit (*Actinidia deliciosa*). Kiwiberry (*Actinidia arguta*) are ample with nutrients, such as dietary fiber, vitamins, mineral elements, organic acids, and many biologically active components [3]. Using spectral analysis, these authors identified ten succinic acid derivatives, six citric acid derivatives, and phenolic acids (eleven quinic acid derivatives, two shikimic acid derivatives) and isolated nine new bioactive compounds, argutinosides A-I. Unlike the common kiwifruit, the kiwiberry has high-frost hardness (down to −30 °C) and a relatively short vegetation period, which potentially allows it to be planted in colder climates [2]. *Actinidia arguta* has fruits of grape size with thin, edible skin containing polyphenols and other secondary metabolites [4,5]. The kiwiberry can be consumed whole, which increases its nutritional value. *Actinidia arguta* cultivars are highly nutritious, low-calorie fruits with the potential to deliver a range of health benefits [1,6,7,8,9,10,11]. They are good source of vitamins, especially vitamin C, and polyphenols, are better than apples and oranges [6,7], and show high activity of cysteine protease and actinidin, which promote digestion and laxation in the human body [12]. On the other hand, actinidin is an allergenic protein in which acidic and basic isoforms were identified in *A. deliciosa* ‘Hayward’ and *A. arguta* ‘Hortgem Tahi’ [13]. Depending on the cultivar, hardy kiwifruits are approximately 3–5 cm long, elliptical, and slightly flattened. Fruit weight ranges from 5 to 20 g and can vary in color from green to blush red or Bordeaux red [14]. There are many cultivars among *Actinidia*, for example Issai, Weiki, Ken’s Red, Miss Green, Jumbo, Takaka Green, Marju Red, and Ananasnaja. Some of them are already commercially cultivated and popular among consumers because of their taste and their appearance [15,16]. Weiki and Ananasnaja are very similar to each other with green flesh and with elongated and slightly flattened shapes. The skin color is green with red blush [7]. The M1 fruit is round with green skin and flesh and contains on average 185.5 seeds. It has a higher content of vitamin C (78.8 mg·100 g^−1^ FW) than Ananasnaja (67.4 mg·100 g^−1^ FW) [15]. Ananasnaja, also called Anna, is very similar to the Weiki and Miss Green cultivars with an elongated and slightly flattened shape [7,15]. It has a size similar to the Bingo cultivar but is less flattened, and its base color is dark green, and the blush is cherry-red [10]. Geneva is ball-shaped and slightly flattened. The skin color is green with a light red blush [7]. Jumbo is longer than Bingo, strongly flattened, and completely green with no blush [10]. Latocha [7] compared seven cultivars of *A. arguta* and concluded that the biggest fruit is the cultivar Jumbo with an average weight 10.74 g but has the lowest level of vitamin C (7.0 mg·100 g^−1^ FW). Bingo is an F1 hybrid between *A. arguta* var. *purpurea* ‘Purpurna Sadova’ and *A. arguta* but has dominant characteristics of *A. arguta*, which is why nowadays it is classified as *A. arguta*. The average weight of the fruit is 8.1 g to about 14 g and contains about 175 yellow-brown seeds. It is elliptical and clearly flattened. The ripe fruit is not astringent, has a smooth, yellow-green skin with an intense reddish-pink blush [10]. Latocha et al. (2010) [17] showed that hardy kiwifruits contain higher amounts of phenolic compounds than fruits of *A. deliciosa* ‘Hayward’. Nishiyama [8], Latocha [7], Latocha and Krupa [15], and Bieniek [9] have indicated that the levels of vitamin C in *A. arguta* fruits are not constant and depends on many factors, such as the growing conditions, sun exposure, and their genotype. The nutritional value of the hardy cultivars depends on mineral composition, polyphenols, vitamins, the total quantity of dietary fiber, the ratio of insoluble to soluble fraction, and actinidin content [5]. Some authors indicate that the mineral composition of *Actinida* fruits may also differ between cultivars and depend on soil and climatic conditions, fertilization and irrigation, and harvest dates [6,11,15,18]. The mentioned bioactive compounds may interact with other nutrients and change their bioavailability.

There are 2.3% to 6.4% of mineral elements in the bodies of mammals. They can be divided according to their contents in the body into at least two groups: macroelements and microelements (trace elements). Kations, such as calcium, sodium, potassium, and magnesium, belong to macroelements; the other kations belong to microelements: iron, manganese, zinc, and cooper. All of them are considered essential components because they play important roles in body metabolism. Macroelements found in extracellular fluids, intracellular structures, and cell membranes play an essential role in such vital functions as nerve conduction, muscle contraction, and membrane permeability. Trace elements are constituents of proteins (e.g., haemoglobin), and hundreds of enzymes are involved in most major metabolic pathways. They should be consumed in the adequate amounts [19] but not higher than tolerable intake levels [20]. Mineral elements can interact not only among themselves but also with other food components on the principle of synergism or antagonism. Some studies have reported that the kiwifruit could be used as a dietary supplement, especially for individuals with hypercholesterolemia and cardiovascular disorders [2,21]. Mortality statistics shown that cardiovascular diseases (45% of all deaths) remain the most common cause of death in Europe [22]. It is well known that dietary fiber lowers blood cholesterol by binding bile acids and then excreting them. The dietary fiber content in the *Actinidia arguta* fruit is higher than in the most popular cultivar, Hayward of *A. delicosa* [23]. Moreover, kiwifruit is rich in potassium [24], an important macroelement in managing high blood pressure. Potassium lessens the effects of sodium, and the more sodium is lost through urine. Hypertension affects the heart and accelerates the rate of formation of the atherosclerotic plaques.

Antioxidative, antimicrobal, anti-inflammatory, neuroprotective, and antiallergic activities of *Actinidia arguta* in vitro and in vivo were shown [4,5,25]. Until now, however, there are no studies on the mineral profiles in animal models, taking into account the bioavailability and the circulation of mineral elements in the trophic chain. The aim of this study is to presents the influence of consumption of fruits of six *A. arguta* cultivars on the mineral profile and bioavailability of macro- and microelements in rats with induced hypercholesterolemia. We suppose that kiwiberry, which are consumed with the peel (mainly insoluble dietary fiber), together with exogenous cholesterol could change the bioavailability of the essential elements, Ca, Mg, Fe, Mn, Zn, and Cu, in rats as an animal model. Knowledge of macro- and microelement bioavailability is needed to translate physiological requirements into actual dietary requirements when elevated TG, TC, and LDL-C in the human population become common.

## 2. Materials and Methods

### 2.1. Animal Housing and Experimental Diets

The trial was performed at the research facilities of the Department of Physiological Sciences Warsaw University of Life Sciences (SGGW). Male Wistar rats (114 ± 10 g) were randomly classified into eight groups (each of seven rats) and fed semipurified diets. All animals were submitted to an adaptation period of five days and fed the control diet. The control diet included (g/kg): casein (150), soybean oil (100), cellulose (10), vitamin (10) (AIN-93-MX Mineral mix Cat. No. 960 402), mineral mixtures (36.7) (AIN-93-MX mineral mix Cat. No. 960 400) of the American Institute of Nutrition for laboratory animals, choline (2), and wheat starch added up to 1 kg (691.3). They were housed in individual plastic cages (TECNIPLAST S. p. A, 21 020, Italy), and clean water and feed were provided ad libitum for six weeks of the experiment. Rats were fed once daily at 10:00 am. The feed intake was controlled daily. The first group (control) was offered the basic diet, and the second positive control group (chol) was hypercholesterolemic (containing 1% of cholesterol—Sigma-Aldrich-C8667) without inclusion of kiwiberry. The other experimental groups contained both cholesterol (1%) and 5% of kiwiberry of different cultivars: Bingo/chol, M1/chol, Anna/chol, Weiki/chol, Jumbo/chol, and Geneva/chol. At the end of the experiment (after 24 h of starvation), rats were anesthetized using inhalation of halothane (Narcotan-Zentiva). The study procedures were approved by the Animal Care Committee of Warsaw University of Life Sciences (SGGW), Poland.

### 2.2. Actinidia arguta Samples

Hardy kiwifruits (*Actinidia arguta* (Siebold et. Zucc) Planch. ex. Miq.) were grown on the ecological, non-fertilized field of the Department of Environmental Protection, Warsaw University of Life Sciences (SGGW), Poland. Six cultivars were studied: Bingo, M1, Anna (Annasnaja), Weiki, Jumbo, and Geneva. Fruits were picked at their eating ripeness stage from different parts of vines in 2013. Harvested fruits (3 kg of each cultivar) were washed under tap water and then freeze-dried with the peel and added to the rat diets.

### 2.3. Mineral Analyses of Soil Samples

The soil was sieved through a sieve (1 mm^2^). The soil samples were then weighed into quartz vessels and burnt in a muffle furnace with temperature control at about 480 °C for 4–6 h. After cooling, the powder was quenched with 20% HCl and refluxed in a heating block for 60 min at 148 °C. At the end, the samples were filtered through a hard filter washing with small portions of acidified water to a volume of 50 mL. Mineral analyses were performed using the atomic emission spectrometer inductively coupled plasma atomic emission spectroscopy (ICP-AES) Thermo iCAP 6500 DUO. The method consists of measuring in test solutions the intensity of emitted radiation, which is a measure of the concentration of the mineral determined. Pure argon (99.9%) was used as the carrier gas. For Ca, the average of three lines with lengths of 315.8, 373.6, and 422.6 nm was used. For Mg the average value from the lines, 279.5, 285.2, and 382.9 nm, was used. For Fe, the average value from the lines, 238.2 and 259.9 nm, was used. For Mn, the mean value from the lines, 257.6, 260.5, and 293.9 nm, was used. For Zn, the mean value from the lines, 202.5 and 213.8 nm, was used. For Cu the average value from the lines, 224.7, 324.7, and 327.3 nm, was used.

### 2.4. Mineral Analyses of Biological Samples

Approximately 0.5 g of lyophilized fruits, liver, kidney, and spleen were placed in Teflon vessels, and 5 mL of HNO_3_ (Merck 1.00441) and 1 mL of H_2_O_2_ (Merck 1.07298) were added. The samples were mixed and allowed to react for 24 h. Mineralization was carried out in the microwave Milestone Ethos 900 (USA–Italy). The mineral elements, Ca, Mg, Fe, Mn, Zn, and Cu, were determined by flame atomic absorption spectrometry in a Perkin-Elmer 1100 B, using hollow cathode lamps at 422.7, 285.2, 248.3, 279.5, 213.9, and 324.8 nm, respectively. The standards were prepared on the base of Titrisol Standard series (Merck).

For the estimation of the mineral bioavailability, the “three-point assay” model was applied according to the description of Littell et al. [26] This model was used after confirmation that in all groups the correlation between mineral intake and content in the liver was linear (y = a + bx). The relative bioavailability value (RBV) of the mineral was calculated as follows:

$$\mathrm{RBV}=\frac{b\;\left(\mathrm{kiwiberry}\right)}{b\;\left(\mathrm{chol}\right)}\times 100$$

where b (kiwiberry) is the tangent of an angle of regression curve for mineral contents in the livers of rats fed diet with kiwiberry, b (chol) is the tangent of an angle of regression curve for mineral contents in the livers of rats fed chol diet (1% cholesterol).

More details of the calculation of mineral bioavailability have been presented in [27]. 

### 2.5. Statistical Analyses

The results are presented as means ± SD (standard deviation). One-way analysis of variance (ANOVA) for statistical evaluation of results was used and post-hoc Duncan’s new multiple range test was applied (*p* < 0.05). For the bioavailability of minerals, the Scheffe test (*p* < 0.05) was applied.

## 3. Results

It is very important to choose among the market available kiwiberry fruits, the most valuable in terms of nutritional value. Most of the publications concentrate on the bioactive compounds, such as dietary fiber, polyphenols, vitamins, and enzymes [4,28,29]. Our study brings new data on mineral composition in six cultivars of mini kiwifruits and helps to indicate which one of them is the best source of minerals. First, we analyzed soil richness in macro- and microelements. It should be noted that no fertilization was applied on the studied field. The soil pH was between 7.1 and 7.4. Mineral composition of the soil is presented in Table 1. The highest contents of microelements was in the Weiki cultivar fruits and the lowest in the Bingo mini kiwi (Table 1). M1, Anna, Geneva, and Jumbo have comparable amounts of Cu, Mn, Zn, and Fe in whole fruits (together with peel and seeds). 

The mineral element contents in soil and six cultivars of kiwiberry fruits are shown in Table 1.

The Weiki cultivar seems to be the richest source of trace elements, while M1 and Anna cultivars contained the highest level of macroelements. We used the rat model to estimate the mineral profile depending on hypercholesterolemic diets supplemented with various kiwiberries. The mineral contents in the diets for rats are presented in Table 2. Diets supplemented with Anna and Weiki cultivars of kiwiberry differed significantly from the control and chol diets in the Cu, Zn, Mg, and Ca contents. The manganese contents in all experimental diets ranged from 10.7 to 12.8 mg·kg^−1^ DM. The highest contents of Fe were in the diets with 5% of Weiki kiwiberry added. Diets supplemented with kiwiberry fruits were willingly consumed by rats. The feed intakes during the experiment were 687 ± 37, 710 ± 31, 695 ± 30, 708 ± 28, 692 ± 36, 718 ± 15, 719 ± 17, and 718 ± 19 g in the control, chol, Bingo/chol, M1/chol, Ann/chol, Weiki/chol, Jumbo/chol, and Geneva/chol groups, respectively.

The contents of mineral compounds in the livers of rats are shown in Table 3. The contents of manganese in the livers were comparable except for the M1/chol group in which the contents were the lowest (1.7 ± 0.1 mg·kg^−1^ DW). The zinc contents in the livers ranged from 28.5 in the Anna/chol group to 33.3 mg·kg^−1^ DW in the control group. Rats from the Geneva/chol group had the lowest contents of Cu in the livers and significantly differed from the control group. The manganese, copper, and zinc contents in the livers were slightly lower in all groups receiving the diets of cholesterol and kiwiberries compared to the control group (*p* < 0.05). The high content of Fe in the livers was obtained in rats fed diets with M1, Anna, Weiki, Jumbo, and Geneva cultivars of *A. arguta*. There were no significant differences in the Mg contents in the livers. The highest value of Ca was in rat livers from the Anna/chol group. The mineral contents were also determined in the spleens and kidneys of rats (Table 3). The highest contents (*p* < 0.05) of manganese were recorded in the spleens and kidneys in the Jumbo/chol group and in the Weiki together with the Jumbo/chol group, respectively. No significant differences in contents of other minerals in the spleens and kidneys were obtained, except in the Fe contents in the spleens, which were the highest in the M1/chol group (Table 3). 

**Figure 1:**
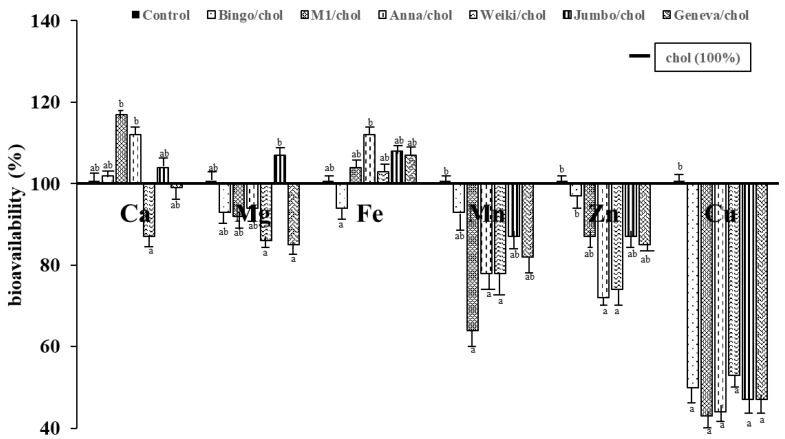
Bioavailability of macro- and microelements in rats fed diets with kiwiberry fruits and cholesterol. ^a,b^—columns marked with different letters differ at *p* < 0.05 (*n* = 7). Abbreviations: control—rats receiving control diet, chol—rats receiving control diet with 1% of cholesterol, the horizontal line (100%), Bingo/chol—rats receiving diet with 1% of cholesterol and 5% of Bingo fruits, M1/chol—rats receiving diet with 1% of cholesterol and 5% of M1 kiwiberry, Anna/chol—rats receiving diet with 1% of cholesterol and 5% of Anna kiwiberry, Weiki/chol—rats receiving diet with 1% of cholesterol and 5% of Weiki kiwiberry, Jumbo/chol—rats receiving diet with 1% of cholesterol and 5% of Jumbo kiwiberry, Geneva/chol—rats receiving diet with 1% of cholesterol and 5% of Geneva kiwiberry.

The bioavailability of the selected minerals is shown in Figure 1. The reference group was the chol group (positive control), which was placed on the graph as 100% (the bolded line on the Figure 1). Supplementation of all studied kiwiberries for the atherogenic diet decreased bioavailability determined on the basis of the concentrations of Mn, Cu, and Zn in the livers vs. control. A significant decrease of manganese, copper and zinc bioavailability was obtained in rats fed atherogenic diets with Anna and Weiki cultivars of kiwiberry addition versus the control rats. The bioavailability of Fe was significant higher in the Anna/chol group compared to the control. In other groups, except for the Bingo/chol group, there was a slight increase in bioavailability of Fe. Only in the Jumbo/chol group an increase in bioavailability of Mg was recorded and was significant versus the Weiki/chol and Geneva/chol groups. The bioavailability of Ca was significantly higher in the M1/chol and Anna/chol groups versus the Weiki/chol group. A slight decrease of Ca bioavailability was also obtained in the Geneva/chol group.

## 4. Discussion

Our results indicate that Weiki is more abundant in the analyzed microelements (Fe, Mn, Zn, Cu) compared to Anna (Table 1) and that is why Weiki should be more widespread and commonly cultivated commercially around the world. Bieniek [16] has shown that fruit of the ‘Sientiabrskaja’ cultivar of *A. arguta* contained the highest concentrations of Ca, and Mg as well as Cu and Mn compared to the hybrid cultivars of *A. arguta* × *A. arguta* var. *purpurea* or *A. arguta* var. *purpurea*. This is also confirmed by our study; *A. arguta* (especially the Weiki cultivar) is a better source of microelements than the hybrids (for example Bingo). Latocha and Krupa [15] indicated that Ananasnaja (Anna) and M1 have comparable concentrations of Ca. These cultivars also have higher contents of Ca than other kiwiberry fruits in our study (Table 1). Okamoto and Goto [6] pointed out that *A. arguta* contains higher Ca and Mg than *A. deliciosa* (cultivar Hayward) and the domestic cultivar of apple, Fuji, which were purchased at a market. *Actinidia arguta* and *A. arguta* var. *purpurea* and their hybrids are recommended as rich sources of Fe, Cu, and Mg by Ferguson and Ferguson [30], Latocha and Krupa [15], Latocha et al. [17], and Bieniek [16]. Latocha [2] suggests that the mineral components in kiwiberry fruits depend more on genetic features of the plant than on growing conditions. Bieniek and Dragańska [11] demonstrated that the concentrations of macroelements (inter alia Ca and Mg) in Ukrainian cultivars of *A. arguta* and *A. arguta* var. *purpurea* significantly depended on the relationships between cultivars and meteorological factors in specific phenophases.

Consumption of kiwiberry fruits provides many health benefits [2,17,21,23]. They might be the result of the interaction of the natural ingredients present in *Actinidia arguta* fruits. Some of these interactions can enhance biological activity of the nutrients or inhibit them. In the study, we focused on the bioavailability of valuable micro- and macroelements. We have already known that *A. arguta* fruits are a precious source of minerals [31] and that kiwifruit supplementation protects the aortas and livers in rats with induced hypercholesterolemia [21]. We are the first to report that diet supplementation with kiwifruit changes the bioavailability of selected micro- and macroelements in rats loaded with cholesterol. Reiland and Slavin [32] underline that more and more evidence suggests that the health benefits of fruits depend on the synergies or interactions of bioactive compounds and other nutrients in whole diets. That is the way we designed the study to test the impact of the whole diet on the mineral profile and bioavailability of selected minerals in rats. Moreover, rats were fed atherogenic diets, and we can indirectly conclude on the impact of the nutrients on the mineral balance. We studied the contents of Mn, Cu, Zn, Fe, Mg, and Ca, and some descriptions of their functions are needed to underline their important roles in the body.

Manganese acts as both a constituent of metalloenzymes and an enzyme activator. The MnSOD is an antioxidant enzyme that contains manganese as a functional component and protects against oxidative injury by catalyzing the dismutation of O_2_^−^. Copper is included in approximately 20 enzymes involved in reduction/oxidation processes. Copper deficiency can be a significant risk factor for diseases related to oxidative reduction homeostasis and lipid metabolism [33]. Zinc plays a catalytic or a structural role in more than 200 enzymes involved in digestion, metabolism, reproduction, and wound healing. Zinc is an antioxidant and anti-inflammatory agent [34]. These elements help to avoid oxidative damages because they could convert potentially dangerous products of the reactive oxygen species. There was a significant decrease in copper bioavailability in rats with hypercholesterolemia-induced dietary supplements of kiwifruit, which may be associated with a high content of dietary fiber in these fruits (*A. arguta* are eaten with the peel) and reduced copper absorption. We have also demonstrated in experiments with rats loaded with cholesterol and organic kiwifruit (*A. deliciosa* cultivar Hayward) [35] a decreased bioavailability of Cu and a significant decrease of Mn and Zn. In the present study bioavailability of Mn and Zn was also lowered (Figure 1). Reduced manganese, copper, and zinc bioavailability (determined on the basis of the concentrations of those elements in kidneys) in hypercholesterolemic rats fed with organic Hayward has also been revealed [18]. This drop in bioavailability could also affect antioxidant defense because Mn, Zn, and Cu in SOD (superoxide dismutase) forms are the first line of antioxidant defense [36]. The disturbance of balance between free radicals and antioxidants leads to oxidative stress and oxidation of LDL cholesterol, which appears to contribute to atherogenesis [37,38]. We have shown that the smallest percentage of lesions in the aortic arch was in the ChGeneva, ChWeiki, and ChAnna, and positive nutritional effects of supplemented *A. arguta* for hypercholesterolemia were noted [21]. Iron is required in numerous essential proteins, e.g., as the heme-containing proteins [39]. Vitamin C increases iron absorption, and kiwi and kiwifruit are precious sources of this mineral [15,18,24]. We obtained that the Fe contents in the livers of rats supplemented with kiwifruit, except for Bingo, were significantly higher than in the control and chol group (Table 3A). These results were reflected in the bioavailability of iron (Figure 1). On the other hand, inhibitors may reduce nutrient bioavailability. An example of competition for the same uptake system is the interaction between calcium and non-heme iron. A significant decrease in Mg bioavailability was shown in the study with organic and conventional kiwifruit Hayward [35]. The present study also confirms this relationship in the case of the kiwiberry (Figure 1). Dietary fiber may impair mineral balance [40,41]. It was shown by Gralak et al. [42,43], revealing that dietary fiber can reduce the absorption of Cu, Zn Fe, Mg, and Ca. It is the indigestible cell wall component of plants, which is considered to play an important role in human diet and health. The inhibitory effect of dietary fiber can also be used advantageously. Soluble fibers have been shown to increase the rate of bile excretion, reducing serum total and LDL cholesterol, decrease pro-inflammatory cytokines, such as interleukin-18, and decrease levels of C-reactive protein [44]. The effect of kiwifruit on the bioavailability of the studied minerals may be connected with the bioactive substances present in fruits (polyphenols, vitamins, dietary fiber, and tannins) or the other elements of diet.

## 5. Conclusions

The mineral composition of kiwiberries varies significantly among the cultivars. The Weiki cultivar has the highest contents of trace elements, iron, manganese, zinc, and copper, compared to the other cultivars. The Bingo cultivar has substantially the lowest contents of manganese, zinc, and copper. Remarkably higher contents of macroelements, calcium, and magnesium were found in the M1 and Anna cultivars. The addition of 1% of cholesterol to the rat diet did not affect the mineral concentrations in the livers, spleens, and kidneys, except for the calcium contents in the livers, which were higher than in rats fed the control diet. The addition of 5% of any studied kiwiberry fruits significantly decreased the bioavailability of manganese, zinc, and copper in most cases. The bioavailability of calcium, magnesium, and iron was not influenced by kiwiberry supplementation into rat diets significantly, although there was some increasing tendency in Fe bioavailability in groups supplemented with all kiwiberries except with the Bingo cultivar. This trend was also noted in Ca bioavailability except for the Weiki/chol group, where the bioavailability decreased. The bioavailability of magnesium showed a downward trend with the exception of the group fed with the Jumbo kiwiberry.

## Figures and Tables

**Table 1:** Mineral element contents in soil and six cultivars of kiwiberry fruits (DM basis); mean ± SD.

	Ca (g/kg)	Mg (g/kg)	Fe (mg/kg)	Mn (mg/kg)	Zn (mg/kg)	Cu (mg/kg)
Soil	8.82 ± 3.35	2.38 ± 1.49	6031 ± 1018	271 ± 79	90 ± 9	15 ± 4
*Actinidia* cultivars						
Bingo	1.12 ± 0.11 ^a^	0.88± 001 ^b^	21 ± 1 ^a^	4 ± 0.2 ^a^	10 ± 1.3 ^a^	5 ± 0.1 ^a^
M1	1.36 ± 0.11 ^b^	1.01 ± 0.03 ^c^	22 ± 1 ^a^	7 ± 0.4 ^b^	14 ± 1.6 ^b^	9 ± 0.4 ^b^
Anna	1.51 ± 0.14 ^b^	0.93 ± 0.02 ^b^	22 ± 1 ^a^	7 ± 0.2 ^b^	14 ± 1.2 ^b^	9 ± 0.3 ^b^
Weiki	1.03 ± 0.10 ^a^	0.90 ± 0.01 ^b^	38 ± 1 ^b^	9 ± 0.3 ^c^	17 ± 1.1 ^c^	11 ± 0.2 ^c^
Jumbo	1.02 ± 0.10 ^a^	0.85 ± 0.01 ^a^	23 ± 1 ^a^	7 ± 0.1 ^b^	14 ± 1.3 ^b^	7 ± 0.2 ^b^
Geneva	1.05 ± 0.08 ^a^	0.81 ± 0.01 ^a^	22 ± 1 ^a^	7 ± 0.1 ^b^	14 ± 0.7 ^b^	7 ± 0.1 ^b^

^a–c^—means of *Actinidia* cultivars in columns marked with different letters differ at *p* ≤ 0.05 (*n* = 5).

**Table 2:** Contents of mineral elements in the diets for rats (mg·kg^−1^ DM); mean ± SD.

Diets/Groups	Ca	Mg	Fe	Mn	Zn	Cu
Control	4707 ± 345 ^a^	485 ± 28 ^a^	76.3 ± 18.3 ^a^	11.3 ± 0.4	35.2 ± 0.6 ^a^	3.7 ± 1.2 ^a^
chol	4591 ± 450 ^a^	514 ± 38 ^a^	80.9 ± 10.2 ^a^	10.7 ± 0.8	37.3 ± 2.0 ^a^	4.5 ± 1.8 ^a^
Bingo/chol	4973 ± 367 ^a^	586 ± 21 ^b^	83.9 ± 9.9 ^a^	11.9 ± 0.7	38.7 ± 3.8 ^a^	6.5 ± 1.5 ^ab^
M1/chol	5431 ± 487 ^b^	580 ± 25 ^b^	88.1 ± 8.7 ^ab^	12.6 ± 1.2	42.6 ± 5.1 ^ab^	7.6 ± 1.0 ^b^
Anna/chol	5721 ± 504 ^b^	615 ± 86 ^b^	88.4 ± 8.1 ^ab^	12.2 ± 0.4	47.6 ± 7.7 ^b^	7.2 ± 0.6 ^b^
Weiki/chol	5500 ± 317 ^b^	646 ± 41 ^b^	91.8 ± 14.4 ^b^	12.8 ± 1.0	49.3 ± 6.9 ^b^	7.6 ± 0.7 ^b^
Jumbo/chol	4873 ± 205 ^a^	499 ± 37 ^a^	83.3 ± 14.1 ^a^	11.9 ± 0.4	38.9 ± 4.9 ^a^	6.5 ± 0.4 ^ab^
Geneva/chol	4892 ± 324 ^a^	659 ± 57 ^b^	81.5 ± 17.3 ^a^	11.7 ± 1.2	40.2 ± 7.0 ^a^	7.1 ± 1.2 ^b^

^a,b^—means in columns marked with different letters differ at *p* < 0.05 (*n* = 5). Abbreviations: control—control diet, chol—control diet with 1% of cholesterol, Bingo/chol—diet with 1% of cholesterol and 5% of Bingo fruits, M1/chol—diet with 1% of cholesterol and 5% of M1 kiwiberry, Anna/chol—diet with 1% of cholesterol and 5% of Anna kiwiberry, Weiki/chol—diet with 1% of cholesterol and 5% of Weiki kiwiberry, Jumbo/chol—diet with 1% of cholesterol and 5% of Jumbo kiwiberry, Geneva/chol—diet with 1% of cholesterol and 5% of Geneva kiwiberry.

**Table 3:** Contents of minerals (mg·kg^−1^ DM) in (A) livers, (B) spleens, and (C) kidneys of rats fed diets with kiwiberry fruits; mean ± SD.

**(A)**						
**Group**	**Ca**	**Mg**	**Fe**	**Mn**	**Zn**	**Cu**
Control	21.2 ± 5.5 ^a^	200 ± 35	113 ± 28 ^ab^	2.7 ± 0.3 ^b^	33.3 ± 2.0	6.5 ± 1.4 ^b^
chol	28.8 ± 4.0 ^b^	201 ± 18	97 ± 16 ^a^	2.5 ± 0.5 ^ab^	31.0 ± 3.2	5.4 ± 1.0 ^ab^
Bingo/chol	21.1 ± 3.5 ^a^	206 ± 13	110 ± 10 ^ab^	2.4 ± 0.3 ^ab^	32.7 ± 3.6	5.6 ± 0.6 ^ab^
M1/chol	27.2 ± 4.1 ^ab^	197 ± 7	121 ± 18 ^b^	1.7 ± 0.1 ^a^	31.7 ± 2.3	4.9 ± 0.7 ^ab^
Anna/chol	31.0 ± 6.0 ^b^	202 ± 15	127 ± 25 ^b^	1.9 ± 0.5 ^ab^	28.5 ± 2.5	5.3 ± 1.0 ^ab^
Weiki/chol	20.2 ± 2.0 ^a^	218 ± 11	135 ± 24 ^b^	2.3 ± 0.3 ^ab^	33.0 ± 1.8	5.9 ± 1.5 ^ab^
Jumbo/chol	21.3 ± 5.0 ^a^	215 ± 12	132 ± 16 ^b^	2.4 ± 0.4 ^ab^	31.5 ± 3.0	5.2 ± 0.9 ^ab^
Geneva/chol	22.0 ± 3.4 ^a^	213 ± 11	131 ± 31 ^b^	2.1 ± 0.3 ^ab^	30.2 ± 1.2	4.5 ± 0.5 ^a^
**(B)**						
**Group**	**Ca**	**Mg**	**Fe**	**Mn**	**Zn**	**Cu**
Control	42.7 ± 13.2	254 ± 14	298 ± 31 ^a^	1.1 ± 0.5 ^a^	28.5 ± 3.5	1.9 ± 0.1
chol	42.0 ± 13.0	252 ± 9	283 ± 71 ^a^	0.6 ± 0.1 ^a^	26.9 ± 2.8	2.1 ± 0.4
Bingo/chol	38.0 ± 8.1	250 ± 9	339 ± 71 ^ab^	1.9 ± 0.8 ^ab^	27.5 ± 1.5	2.1 ± 0.3
M1/chol	39.9 ± 8.3	252 ± 32	377 ± 132 ^b^	1.2 ± 0.8 ^ab^	26.8 ± 3.4	2.2 ± 0.4
Anna/chol	43.1 ± 8.2	247 ± 6	300 ± 104 ^a^	2.3 ± 0.4 ^b^	25.5 ± 1.4	2.1 ± 0.1
Weiki/chol	39.5 ± 9.0	252 ± 21	346 ± 91 ^ab^	2.6 ± 1.2 ^b^	26.6 ± 3.5	2.1 ± 0.3
Jumbo/chol	43.8 ± 11.0	257 ± 20	288 ± 67 ^a^	3.0 ± 0.4 ^b^	28.6 ± 3.2	2.0 ± 0.2
Geneva/chol	37.9 ± 7.4	247 ± 12	298 ± 75 ^a^	0.7 ± 0.1 ^a^	23.3 ± 1.8	1.8 ± 0.2
**(C)**						
**Group**	**Ca**	**Mg**	**Fe**	**Mn**	**Zn**	**Cu**
Control	38.4 ± 10	237 ± 9	71.4 ± 3.9	0.8 ± 0.2 ^a^	26.7 ± 0.9	4.6 ± 0.1
chol	38.6 ± 14	230 ± 8	69.9 ± 10.0	1.0 ± 0.4 ^a^	27.6 ± 2.6	4.8 ± 0.2
Bingo/chol	34.8 ± 7.5	224 ± 11	69.5 ± 3.1	1.0 ± 0.1 ^a^	25.8 ± 1.6	4.9 ± 0.2
M1/chol	35.0 ± 5.0	219 ± 10	72.0 ± 7.0	0.9 ± 0.2 ^a^	26.1 ± 1.9	4.7 ± 0.2
Anna/chol	36.3 ± 6.4	224 ± 8	69.8 ± 6.7	1.3 ± 0.3 ^ab^	27.4 ± 2.0	4.5 ± 0.2
Weiki/chol	36.5 ± 7.0	227 ± 15	69.6 ± 3.7	1.6 ± 0.4 ^b^	26.7 ± 2.1	4.7 ± 0.2
Jumbo/chol	36.1 ± 4.9	226 ± 13	68.2 ± 5.9	1.6 ± 0.4 ^b^	27.0 ± 3.3	4.8 ± 0.3
Geneva/chol	37.3 ± 7.1	224 ± 4	68.6 ± 6.8	1.5 ± 0.3 ^b^	28.7 ± 1.5	4.7 ± 0.2

^a,b^—means in columns marked with different letters differ at *p* < 0.05 (*n* = 7). Abbreviations: control—rats receiving control diet, chol—rats receiving control diet with 1% of cholesterol, Bingo/chol—rats receiving diet with 1% of cholesterol and 5% of Bingo fruits, M1/chol—rats receiving diet with 1% of cholesterol and 5% of M1 kiwiberry, Anna/chol—rats receiving diet with 1% of cholesterol and 5% of Anna kiwiberry, Weiki/chol—rats receiving diet with 1% of cholesterol and 5% of Weiki kiwiberry, Jumbo/chol—rats receiving diet with 1% of cholesterol and 5% of Jumbo kiwiberry, Geneva/chol—rats receiving diet with 1% of cholesterol and 5% of Geneva kiwiberry.

## Data Availability

The data presented in this study are available in this article.
